# Delays in Admittance-Controlled Haptic Devices Make Simulated Masses Feel Heavier

**DOI:** 10.1371/journal.pone.0138023

**Published:** 2015-09-11

**Authors:** Irene A. Kuling, Jeroen B. J. Smeets, Piet Lammertse, Bram Onneweer, Winfred Mugge

**Affiliations:** 1 MOVE Research Institute Amsterdam, Faculty of Human Movement Sciences, VU University, Amsterdam, the Netherlands; 2 Moog B.V., Nieuw-Vennep, the Netherlands; 3 Department of Biomechanical Engineering, Delft University of Technology, Delft, the Netherlands; University of Bologna, ITALY

## Abstract

In an admittance-controlled haptic device, input forces are used to calculate the movement of the device. Although developers try to minimize delays, there will always be delays between the applied force and the corresponding movement in such systems, which might affect what the user of the device perceives. In this experiment we tested whether these delays in a haptic human-robot interaction influence the perception of mass. In the experiment an admittance-controlled manipulator was used to simulate various masses. In a staircase design subjects had to decide which of two virtual masses was heavier after gently pushing them leftward with the right hand in mid-air (no friction, no gravity). The manipulator responded as quickly as possible or with an additional delay (25 or 50 ms) to the forces exerted by the subject on the handle of the haptic device. The perceived mass was ~10% larger for a delay of 25 ms and ~20% larger for a delay of 50 ms. Based on these results, we estimated that the delays that are present in nowadays admittance-controlled haptic devices (up to 20ms) will give an increase in perceived mass which is smaller than the Weber fraction for mass (~10% for inertial mass). Additional analyses showed that the subjects’ decision on mass when the perceptual differences were small did not correlate with intuitive variables such as force, velocity or a combination of these, nor with any other measured variable, suggesting that subjects did not have a consistent strategy during guessing or used other sources of information, for example the efference copy of their pushes.

## Introduction

One can judge the mass from the gravitational force of an object by lifting it. Another way to judge mass is to move (or stop) an object and use the inertial mass. For both ways of judging only the haptic modality is required. However, there are many known studies concerning mass (or weight) perception that show that when visual information is available it is taken into account even when it is resulting in a consistent bias or illusion; the size-weight illusion (Charpentier (1891) as summarized in [1]) and more recently size-mass illusion [2], material–weight illusion [3] and shape-weight illusion [4]. These studies show that not only the forces determine perception of mass, but that many characteristics of an object can influence its perceived mass.

In recent technological developments, haptic systems provide the user with haptic feedback and haptic guidance to make task execution easier and more accurate (e.g. [5–8]). An important issue in the development of these devices is the delay between input from the user and feedback to the user. From a technical perspective delays are detrimental to the stability of the system. Therefore developers try to minimize the delays within the system. Moreover, Tzafestas and Velanas [9] showed that delays deteriorate stiffness perception in a psychophysical experiment and presented an adaptive impedance control that was effective at improving this percept despite the presence of delays. Because there will always be delays between input and output in such systems, it is important to know the perceptual effects of such delays.

From research on time perception and adaptation to delays in various sensory modalities, we know that delays can change perceived stimulus characteristics (e.g. [10, 11]). For example, delayed visual feedback makes people perceive the virtual mass of a manipulandum [12] or haptic device [13] as heavier and the compliance of a virtual spring as lower [14] than they actually are. For stiffness, Pressman et al. [15] showed that when moving in and out a virtual surface without visual information the perceived stiffness of this virtual surface systematically depends on the delay between position and force: when the force lagged the penetration, surfaces were perceived as stiffer, and when the force led the penetration, surfaces were perceived as more compliant. Given the fact that delays between modalities influence mass perception the question arises whether delays within the haptic modality (i.e. different temporal alignment between force, velocity, displacement etc.) also influence mass perception.

There are two ways haptic devices can be controlled: either by impedance control or by admittance control. An impedance-controlled haptic device (e.g. Phantom, Geomagic) determines the forces it generates based on its movement. An admittance-controlled haptic device (e.g. HapticMaster, Moog inc.) uses forces to determine the movement of the device. From these technical characteristics of a haptic device hypotheses can be drafted about the nature of perceptual effects of temporal delays. Imagine an admittance-controlled tele-operation system in which an object has to be pushed aside to reach the goal. A delay induces a conflict: on contact the user perceives a high inertia as the initial push does not move the object, while once it starts moving it accelerates faster than the user would expect based on the initially perceived inertia. When the object does not move, the user thinks the object is heavy and exerts more force to overcome the perceived high inertia. Yet, after the delay the object starts accelerating according to the (delayed) exerted force and its actual virtual mass. Using the force information only for mass perception would then lead to an overestimation of the mass, while using the velocity information would lead to an underestimation of the mass.

In this study was tested whether temporal delays in an admittance-controlled haptic human-robot interaction influence the perception of mass. We used a 2AFC staircase method to find the mass that was perceived equally heavy as a reference mass with an added delay. Furthermore, we analysed on which physical properties humans make their decision on mass perception. Intuitively one would expect that the inertia would be estimated based on the relation between the effort and movement. If so, a delay in admittance-controlled devices in a push-off task would result in an increase in perceptual mass: all effort-related information is fully perceived during push-off while the movement information is not as the object will accelerate further after contact is lost.

## Methods

### Subjects

Fourteen subjects (all right-handed, 4 men, 19–30 years of age) took part in the experiment. All subjects were naive about the purpose of the experiment and were compensated for their time.

### Ethics Statement

The experiment is part of an ongoing research program that has been approved by the ethics committee of the Faculty of Human Movement Sciences of VU University Amsterdam. All subjects gave their written informed consent prior to participation.

### Stimulus and Apparatus

An admittance-controlled haptic device (HapticMaster, Moog inc.) simulated various masses. The delay between the input of the user and the feedback of the device to the user is based on several factors. First, the sample frequency of the HapticMaster is 2.0 kHz, which leads to a maximum delay of 0.5ms. Second, there is a delay in the (force) sensors, which is measured to be ~1ms. On top of that it takes time to send the current to the motor (4-5ms for the current to fully build). Combined with a ‘rise time’ of several ms, the total delay between the input of the user and the feedback of the device is estimated to be ~10ms.

We used the HapticMaster with three values for the (additional) delay (0, 25 or 50 ms). During the experiment the subjects were blindfolded and they wore headphones playing loud white noise, to avoid auditory or visual cues to influence the haptic percept. Throughout the experiment the HapticMaster registered the position of the handle and the forces exerted to it (at 2.0 kHz).

### Procedure

**Fig 1:**
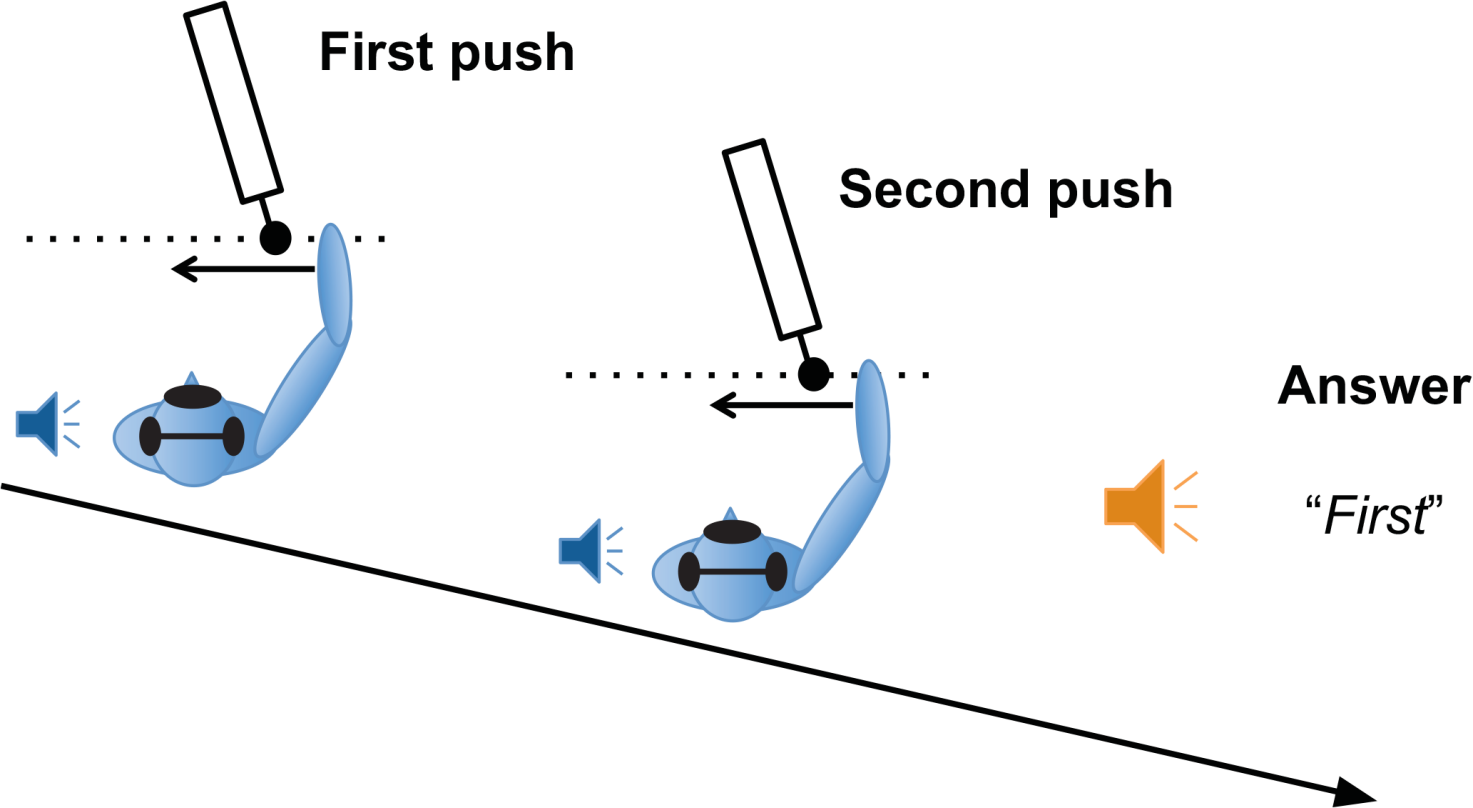
Schematic representation (not to scale) of the task and experimental procedure. The haptic device started ~30 cm in front of the subject’s torso, and 10 cm to the right of the body’s midline. Subjects were blindfolded and were wearing headphones. At a beep the subject pushed the handle gently to the left. After the push the haptic device moved back to the start position, at a second beep the subject pushed again and judged which of the two felt the heaviest.

Subjects were verbally instructed to gently push the HapticMaster leftward with the right hand twice and judge which of the two virtual masses was heavier. Subjects started the experiment with their hand next to their body. When the handle of the HapticMaster moved to the start position (approximately 30cm in front of the subjects body and 10cm to the right of the body’s midline), the subject had to find the handle and push the handle to the left upon a ‘push’-beep (beep-sound of Matlab (Mathworks Ltd., USA)) through the headphones (Fig 1). Subjects were instructed to push the handle in a similar way as pushing someone on a swing, which means: do not hit the handle, and do not guide it along the movement. After the push, the subject withdrew the hand a few centimetres from the start position, and the HapticMaster stopped the handle on a random position between 10 and 20 cm after the start of the movement to avoid any cues about the timing and duration of subsequent pushes. Then the HapticMaster moved the handle back to the start position, changed the characteristics of the virtual mass and a second ‘push’-beep was played, cueing the subject to push again. Because the handle stopped at random end positions, the duration of the push + return (on average 5 seconds) was variable, and independent of the simulated mass, the delay and the characteristics of the push. After the second push, an ‘answer’-beep (100 ms, 660 Hz) was played, and the subjects said out loud which of the two masses they perceived to be heavier. The experiment leader recorded the answer and the next trial was started (Fig 1).

A staircase method (1-up/1-down, step-size 0.25 kg) was used to find the test masses (no additional delay) that perceptually corresponded with the reference mass of 6.0 kg with one of three delays. A staircase was ended after 12 reversals. For each of the three delays for the reference mass, two staircases were executed; the test mass in one staircase started at 3.0 kg and in the other at 9.0 kg. The in total 6 staircases per subject were presented interleaved in a semi-random order: we presented sets consisting of one trial of each staircase that did not yet end. If a staircase was finished, the randomization was run with the remaining staircases. The order of presentation of the reference and comparison masses was fully randomized. On average subjects performed 183 trials and took 45 minutes to complete the experiment. For one of the subjects erroneously a different mass (5kg) was presented with 25ms delay. We therefore excluded the values for this delay for this subject.

### Analysis

**Fig 2:**
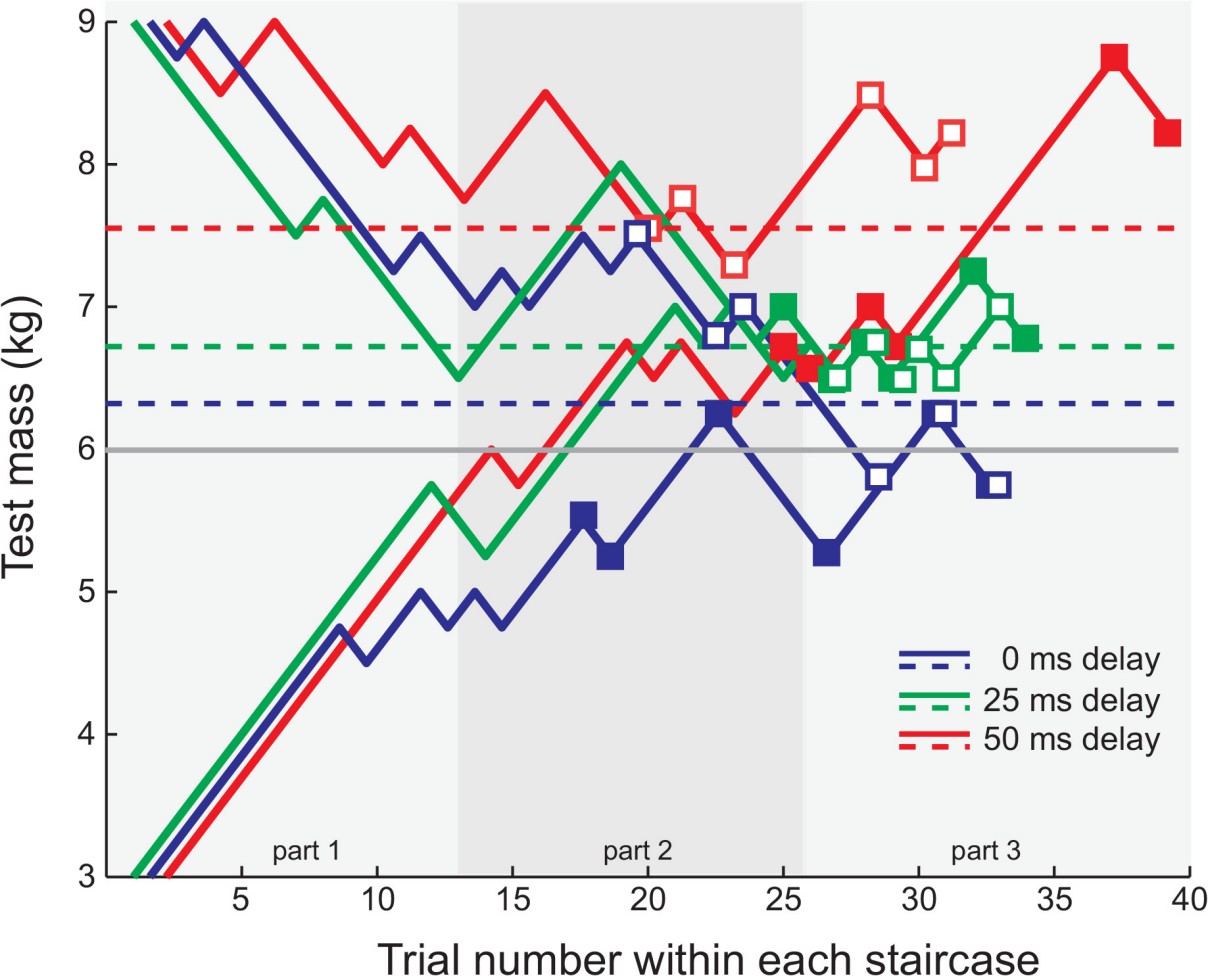
Staircases for an example subject, showing how our analysis method deals with data that is not optimal. For each delay (0ms; blue trace, 25ms; green trace, 50ms; red trace) the data converge towards the perceived mass (dashed lines), calculated based on the last 6 reversals of the two staircases (open squares are the reversals from the staircases from above and filled squares from below). The data of the 50 ms delay (red) converged later than the others and the two staircases of the 50 ms delay reached their stabile value at different moments (trial numbers). For 0ms additional delay the perceived mass is 6.2kg, which is close to the reference mass (thin grey solid line). For 25ms additional delay and 50ms, the perceived masses increase to 6.7kg and 7.5kg respectively. The blue and red curves are shifted slightly rightwards for clarity. The light grey background colours indicate the subdivision into three parts used in the component analysis (see text for details).

For each subject, the perceived mass was calculated for each of the three delays by taking the mean of the test masses presented at the last six reversals of each of the two staircases for that delay (Fig 2). With a one-way RM ANOVA we tested for an effect of delay on the perceived mass.

Adding a delay to the simulated mass changes many aspects of the movement. In order to determine on what information subjects based their judgements, we did a component analysis. Similar to the study of Pressman et al. [15], in which they studied the perception of delayed stiffness, we used various measures to try to explain the judgments of the subjects. We determined various measures based on the force and position data of the individual pushes and tested whether they corresponded with the decision of the subject. The start and end points of the individual pushes were defined as the first samples relative to the peaks in the force data (in backward and forward direction, respectively) at which the force signal had crossed zero.

For each push, the integrated force (F_int_), mean force (F_mean_) and peak force (F_peak_) were calculated and the difference between two subsequent pushes was compared with the answer of the subject. These force measures were chosen because they represent a value that subjects could possibly use to determine mass. The integrated force for example, is a measure of the total force that the subject exerted on the handle to get the mass to move. The same comparison between subsequent pushes was done for several velocity measures, for example the end velocity represents the velocity of the mass at the moment the subjects stops pushing. This ‘push-off’ velocity could potentially be very informative with respect to the mass judgement. We used the mean velocity (v_mean_), peak velocity (v_peak_) and end velocity (v_end_) of the pushes, as well as the duration of the pushes, the distance that was moved during the push and several combinations of these measures: F_peak_/v_peak_, F_mean_/v_peak_, the ratio F_int_/distance (as a measure of integrated force divided by integrated velocity), peak power (P_peak_, the maximum F*v), work (F_int_*distance), and mean acceleration (v_end_/duration). The combination measures represent combined sources, for example to calculate a mass with Newton’s second law of motion we need both force and acceleration. As humans do not perceive acceleration directly, it is more likely that a combination of force and velocity is the best way to predict the subject’s perceived mass.

For all measures we calculated the percentage of ‘largest chosen’. This is the percentage of the trials in which the subject choose the push with the largest value of this measure as being the heavier mass. If subjects would systematically base their decision on one of the measures its percentage ‘largest chosen’ would deviate significantly from chance level (100% is always largest chosen, 0% is always smallest chosen). The percentage of decisions that corresponded with the differences in the measures gives information about the information used for inertial mass perception. It can be expected that many measures will show a correlation with the responses when the differences in the perceived masses are large (i.e. subjects have to exert more force on an object that is heavier). As the perceptual difference between test and reference becomes smaller during the execution of the staircase, subjects will start to guess, and thus correlations will become weaker, and the number of correlated measures will decrease. We therefore divide the experiment in three parts with an equal number of trials (background colours in Fig 2). We will regard the measure(s) that correlate with the subjects’ choices in all three parts of the experiment as the measures that underlie mass perception.

## Results

**Fig 3:**
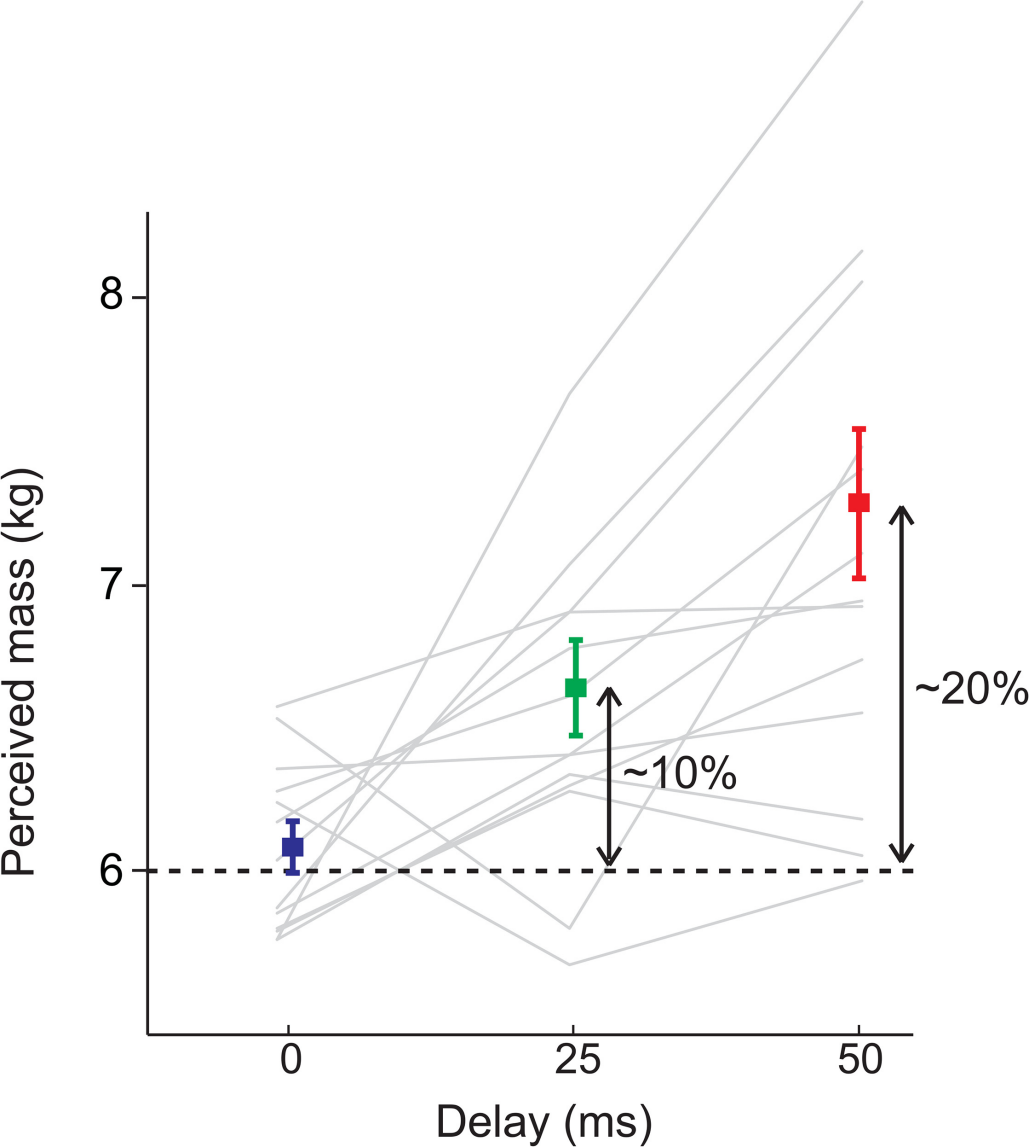
Results. The grey lines show the perceived mass based on the last six reversals of the staircases for each subject. Most subjects show an increasing trend. The coloured squares show the mean perceived mass for the delays. Error bars show standard errors of the mean.

For all subjects, the staircases from above and below more or less converged for all delays, confirming that also with a delay between input and output, subjects can consistently perceive mass. Fig 3 shows that the additional delay causes an increase in perceived mass (~10% for 25ms delay and ~20% for 50ms delay). The RM ANOVA showed a significant effect of delay on perceived mass (F_2,24_ = 11.9, p = .001). Post-hoc pairwise comparisons show significant increases in perceived mass between all delays (0ms vs. 25ms, p = .03, 0ms vs. 50ms, p = .002 and 25ms vs. 50ms, p = .005).

**Fig 4:**
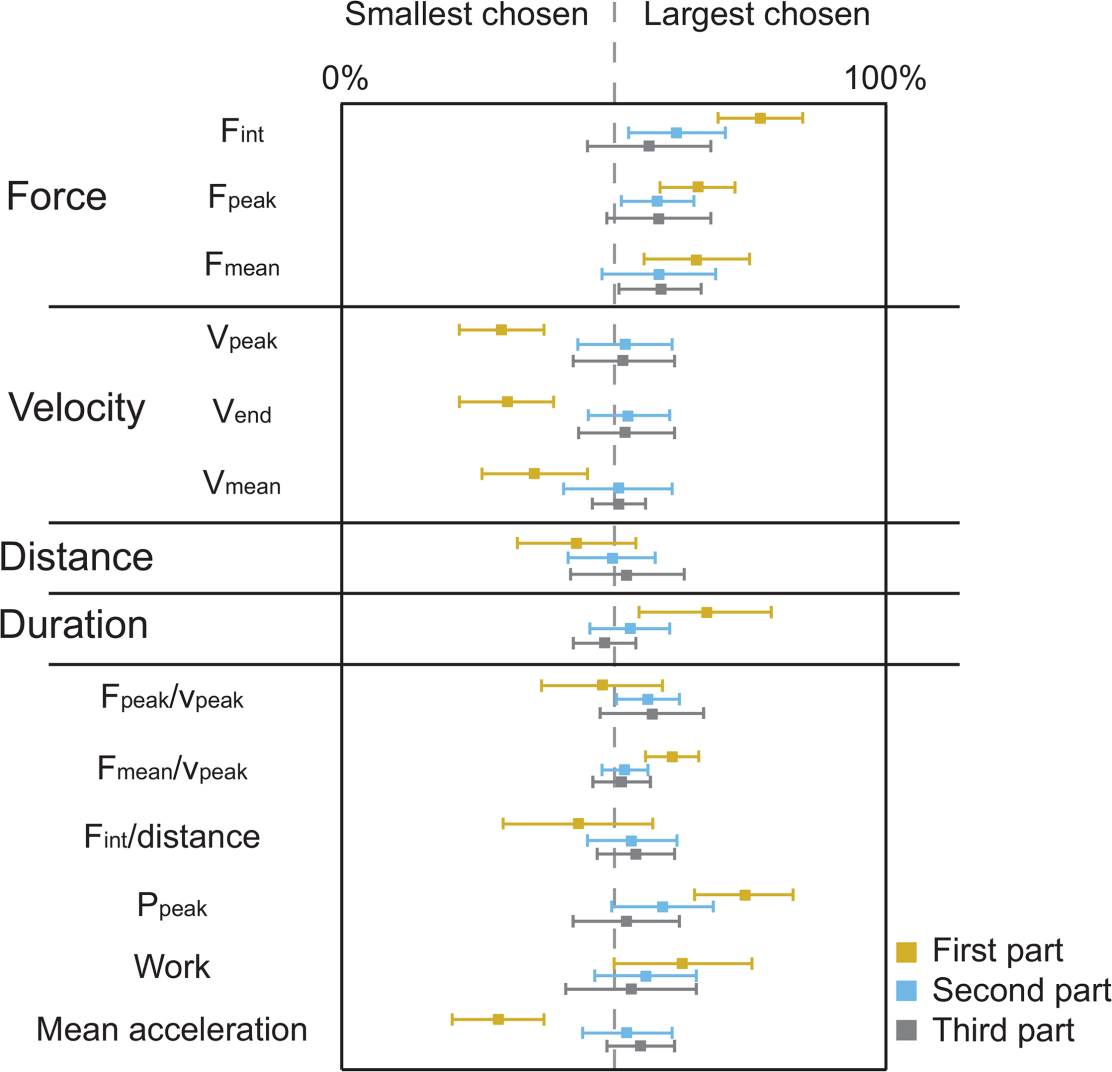
Results of the component analysis. For each measure the percentage of trials in which subjects choose the largest (on this metric) as being the heavier one is shown for the first, second and last part of the trials. Error bars indicate the SE’s. In the first part many measures correlate, but when in the third part the perceptual differences between the test and reference mass are small, there are no longer any significant correlations with the subjects’ decisions.

In the introduction, we argued that using a measure based on the relation of effort and movement would predict an increase of perceived mass with additional delay in an admittance-controlled haptic device. To test which measure is the best explanation of the subjects’ judgements, we analysed the components of information that subjects based their decisions on during the experiment. The mean percentages over subjects with standard deviations can be seen in Fig 4. As expected, many measures showed a clear correspondence with the decisions in the first part of the experiment. In later parts there are no longer significant correspondences.

## Discussion

In this study of gently pushing a virtual mass we showed that delays between the input of the user and feedback of the device to the user in an admittance-controlled device increased the perceived inertial mass of an object. The perceived mass increased ~10% with a delay of 25ms and ~20% with a delay of 50ms.

To maintain stability at the lowest values of the staircases a reference mass of 6kg was used, which is relatively high for tasks with these types of haptic devices. How large the effect of delays would be on lighter virtual masses is still unknown and should be tested in other (lighter) devices. Predicting the results based on the Weber fraction, a principle known in mass perception, it is likely that the fraction of the effect and the mass is more or less constant. The Weber fraction of mass is about 10% for inertial mass [16–18], which is more than twice the step size in our experiment. This might explain why not all staircases for all subjects fully converged within the range of the experiment.

Similar to the study of Pressman et al. [15], in which they studied the perception of delayed stiffness, we used various measures to explain the behavior and the mass judgments of the subjects. In their study the peak force/penetration ratio gave the best predicting results (although not very strong). In our component analysis, we found that the decision of the subjects in the second and third part of the trials was also hard to predict from the exerted force and resulting movement using force, velocity, duration and distance measures and we did not find measures that significantly correlated with the decision of the subject when the perceptual differences were small. In the first part we found correlation between mass judgments and the force measure (larger force led to higher chance of being chosen as the larger mass) and the velocity measures (smaller velocity led to higher chance of being chosen as the larger mass). Combining the two to peak power also showed a strong correlation, as well as the mean acceleration during the push. The effect are not independent and probably disappeared with smaller perceived difference because subjects were either matching them (without delay) or because the conflict between force and velocity measures led to more noisy judgements and guesses. However, the staircase data still showed converging patterns in the second part, which excludes complete guessing.

The list of measures to predict the subjects’ percept is not exhaustive, but it will be hard to give physical meaning to higher-level measures than the chosen ones. In addition to guessing by the subjects, one other explanation why we cannot predict the decision of the subjects very well is that the percept differs from the physical measures. This can be due to noise, or it might be that the subjects use their efference copy of the push. It has been suggested that the subjects’ percept is a combination of information perceived by sensory feedback and the information from the efference copy that gives subjects information about their own action [19]. We have only access to the physical measures, not to the full percept of the subjects. This limits our ability to predict the decisions. Especially when the masses are subjectively (close to) equal, the information from the efference copy and noise might play an important role in the decision.

The delays we used in this study were much larger than the maximum delays that are used (and allowed to maintain stability and safety) in haptic devices, which are in general up to 10-20ms. If we interpolate our results on mass perception linearly, 10 ms delay in the device would mean an increase in mass of 4%, which is probably not noticeable by humans.

One might think that the effect of delays could be taken advantage of to enlarge the range of displayed masses on haptic devices. However, there might be some problems in doing so. First, we would only have a very limited effect because we can only make the perceived mass heavier and we can reach only a 10% mass change. Second, the effect size seems to differ over subjects, which implies that it would be very hard to create a stable mass percept over multiple users. Third, we expect that longer exposure to the presented delays might result in adaptation. Another problem with using delays to increase the perceived mass is the stability of the system and conflicts in tasks in which subjects have to hold the handle continuously. Therefore, we do not think that varying delays would be efficient to enlarge the range of displayed masses on haptic devices.

To conclude, we found that small delays in an admittance-controlled device increase the perceived mass of the simulated object’s virtual mass. This result is in line with the proposal that the human user perceives small delays as changes in the characteristics of an object instead of delays.

## Supporting Information

S1 Appendix Data of subject 1–7. (ZIP)

S2 Appendix Data of subject 8–14. (ZIP)

S3 Appendix Mass values of all subjects. (ZIP)
